# Bacteriocin-Producing *Enterococcus faecium* OV3-6 as a Bio-Preservative Agent to Produce Fermented *Houttuynia cordata* Thunb. Beverages: A Preliminary Study

**DOI:** 10.3390/foods12193520

**Published:** 2023-09-22

**Authors:** Thiwanya Choeisoongnern, Chaiyavat Chaiyasut, Bhagavathi Sundaram Sivamaruthi, Netnapa Makhamrueang, Sartjin Peerajan, Sasithorn Sirilun, Phakkharawat Sittiprapaporn

**Affiliations:** 1 Neuropsychological Research Laboratory, Neuroscience Research Center, School of Anti-Aging and Regenerative Medicine, Mae Fah Luang University, Bangkok 10110, Thailand; 2 Innovation Center for Holistic Health, Nutraceuticals and Cosmeceuticals, Faculty of Pharmacy, Chiang Mai University, Chiang Mai 50200, Thailand; chaiyavat@gmail.com (C.C.); sivamaruthi.b@cmu.ac.th (B.S.S.); 3 Office of Research Administration, Chiang Mai University, Chiang Mai 50200, Thailand; 4 Department of Pharmaceutical Sciences, Faculty of Pharmacy, Chiang Mai University, Chiang Mai 50200, Thailand; 5 Health Innovation Institute, Chiang Mai 50200, Thailand

**Keywords:** bio-preservative, bacteriocin, lactic acid bacteria, *Enterococcus faecium*, fermented plant beverage, *Houttuynia cordata* Thunb.

## Abstract

Microbial contamination affects the quality of the fermented *Houttuynia cordata* Thunb. (*H. cordata*) beverage (FHB). The present study aimed to assess the bio-preservative property of *Enterococcus faecium* OV3-6 (*E. faecium* OV3-6) during the production of FHB. The antimicrobial activity against *Escherichia coli*, *Salmonella*, *Bacillus cereus*, and *Staphylococcus aureus* and the survival of *E. faecium* OV3-6 were studied. Then, FHB fermentation was performed with different preservatives (non-preservative, *E. faecium* OV3-6, cell-free supernatant of *E. faecium* OV3-6, and nisin) with and without representative pathogens. The maximum antimicrobial activity against *S. aureus* and *B. cereus* was observed after 18 h of cultivation in an MRS medium. *E. faecium* OV3-6 was used as a starter to produce the FHB, and the strain survived up to 48 h in the fermented beverage. *E. faecium* OV3-6 and its cell-free supernatant inhibited the growth of *E. coli*, *Salmonella*, *B. cereus*, and *S. aureus* in the stimulated FHB. The non-preservatives and nisin-containing FHB showed inhibition against Gram-positive pathogens. The FHB treated with *E. faecium* OV3-6 was rich in lactic acid bacteria, and the product was at an acceptable level of pH (less than 4.3). Certain limitations were identified in the study, such as lack of nutritional, metabolomics analysis, and safety and consumer acceptability of FHB. The results suggested that *E. faecium* OV3-6 could be used as a bio-preservative to produce fermented plant beverages (FPBs).

## 1. Introduction

Safety and product quality are the major concerns of fermented beverages. Preferably, FPBs are prepared through the lactic acid bacteria (LAB)-mediated fermentation process [1]. FPBs are consumed in large quantities in Asian countries [2,3]. Many studies have reported the benefits of FPB consumption, including disease prevention, cognitive improvement, and anti-aging [2,4,5]. FPBs are prepared using various raw materials such as fruits, vegetables, cereals, and herbs [3]. *Houttuynia cordata* Thunb., known as Plu-Khao in Thai, is a popular plant used to produce FPBs, mainly because of its bioactive properties [6,7]. The reports proved that *H*. *cordata* has several pharmacological activities, including antiviral [8], antibacterial [9,10,11], antioxidant [12], anti-inflammatory [13], and anticancer [6] activities.

The traditional fermentation process (natural fermentation without preservatives or starter cultures) has also been used to produce some FPBs. During fermentation, microbial contamination can occur, which causes spoilage. Many standard techniques, such as thermal process and chemical preservation, have improved the microbial safety of FPBs and increased their shelf life [14,15]. However, these techniques have disadvantages, including altering nutritional values, residual toxic substances (e.g., sulfites), and changes in the taste of the product [16].

LABs act as probiotics and aid in preventing microbial contamination via antimicrobial agents like bacteriocin [17]. Several bacteriocin-producing LAB strains have been reported, such as *Enterococcus faecium* [18,19], *Lactobacillus rhamnosus* [20], *Lactobacillus plantarum* [21,22], *Lactococcus lactis* subsp. *lactis* (*L. lactis*) [23,24], and *Leuconostoc citreum* GJ7 [25]. LAB starters could act as bio-preservatives in fermentation [26].

Nisin, produced by *L. lactis*, is a well-studied bio-preservative commercially used as an additive in the food and beverage industry [27,28]. A recent study stated that nisin could inhibit Gram-positive bacteria [29,30]. In addition, nisin is safe for consumption and easily destroyed by human digestion [29]. However, nisin is limited by its specific usage for acid food and low heat resistance under neutral pH [31].

Previously, we have reported the safety and probiotic properties of the bacteriocin-producing *E. faecium* OV3-6 [32]. A detailed report on the bio-preservative nature of *E. faecium* OV3-6 is not available. Knowing the efficiency of *E. faecium* OV3-6 is essential to ensure that it is a bio-preservative agent to produce FHBs. Therefore, the primary objective of this study was to evaluate the efficiency of *E. faecium* OV3-6 as a bacteriocin-producing bio-preservative bacteria to produce FHBs.

## 2. Materials and Methods

### 2.1. Microbial Strains Used in the Study

*E. faecium* OV3-6 (GenBank accession no: MN453594) isolated from the FHBs was used in the study. *Staphylococcus aureus* CIP 76.25 was obtained from the Nantes-Atlantic National College of Veterinary Medicine, Food Science and Engineering, Nantes, France. *Bacillus cereus* ATCC 11778, *Escherichia coli* ATCC 25922, and *Salmonella* Typhi DMST 22842 were obtained from the culture collection of the Faculty of Pharmacy, Chiang Mai University.

### 2.2. Plant Used in the Study

The *H. cordata* plant was obtained from a farm in Chiang Mai, Thailand. The plant was identified with the help of plant specimens (number: 023239) of the Faculty of Pharmacy, Chiang Mai University.

### 2.3. Antimicrobial Activity of E. faecium OV3-6

An overnight culture of *E. faecium* OV3-6 strain was inoculated into de Man Rogosa and Sharpe (MRS) broth medium (HiMedia, Mumbai, India) and cultivated with moderate stirring at 37 °C for 48 h [33]. The cell-free supernatants (CFS) were collected every 6 h of the culturing process. The antimicrobial activity of *E. faecium* OV3-6 was determined using an agar well diffusion assay [32]. The occurrence of a clear zone around the well was observed in the optimal growth condition of indicator strains (*B. cereus* ATCC 11778 and *S. aureus* CIP 76.25), which were cultured in Brain Heart Infusion (BHI) medium (HiMedia, Mumbai, India). The antimicrobial activity was stated in arbitrary units per milliliter (AU/mL) [32].

### 2.4. Assessment of E. faecium OV3-6 Growth in MRS Medium

*E. faecium* OV3-6 strain was cultured in MRS broth as detailed (refer to Section 2.3). The number of bacterial cells was estimated using optical density (OD) measurement [34]. The samples (at 0, 6, 12, 18, 24, 30, 36, 42, and 48 h) were analyzed with a multi-mode microplate reader (model Beckman Coulter DTX 880, Fullerton, CA, USA) at a wavelength of 595 nm.

### 2.5. Assessment of pH Changes during E. faecium OV3-6 Growth

*E. faecium* OV3-6 strain was cultured in MRS broth as detailed (refer to Section 2.3). The pH changes in the samples (at 0, 6, 12, 18, 24, 30, 36, 42, and 48 h) were measured using a pH meter (model HQ40d, Hach, Loveland, CO, USA) at room temperature.

### 2.6. Survival of E. faecium OV3-6 in the Fermented Beverage (FB) with and without H. cordata

The starter culture (*E. faecium* OV3-6) was cultured in MRS broth at suitable conditions (with maximum antimicrobial activity) obtained from the above experiment.

Two fermentation batches (with *H. cordata* and without *H. cordata*) were performed to study the viability of *E. faecium* OV3-6 and its bio-preservative properties. 1: The FB without *H. cordata* was a mixture of sugar cane and water at a ratio of 1:11 (*w*/*v*). 2: The FB with *H. cordata* was prepared by mixing *H. cordata*, sugar cane, and water at 1:1:10 (*w*/*w*/*v*) [35,36]. The mixtures were pasteurized, and *E. faecium* OV3-6 was inoculated (10%) as a starter for the fermentation process. The fermentation process was conducted at 30 ± 2 °C for 48 h. The MRS broth with starter culture (*E. faecium* OV3-6) was used as a control. The bacterial load of the samples was determined using the plate count method on MRS agar [37], and the results were reported as log colony forming unit per milliliters (log CFU/mL). 

### 2.7. Assessment of the Bio-Preservative Property of E. faecium OV3-6 in Simulated FHB

FHBs were prepared with or without pathogens and preservatives (*E. faecium* OV3-6, CFS of *E. faecium* OV3-6, and nisin). No preservatives or 10% (*v*/*v*) *E. faecium* OV3-6 or 10% CFS of *E. faecium* OV3-6, or 5 mg/kg nisin of the sample, were added to the FHBs. 

Regarding pathogens, a pathogen mixture containing *E. coli* ATCC 5922, *S. typhi* DMST 22842, *S. aureus* CIP 76.25, and *B. cereus* ATCC 11778 was used. The pathogens were cultured in Brain Heart Infusion (BHI) medium (HiMedia, Mumbai, India) at 37 °C for 18 h. The culture was serially diluted in phosphate buffer saline (PBS), and cell count was determined using the plate count method [38,39]. Then, the appropriate dilatation with 10^7^ CFU/mL of pathogen was selected for the study, and the pathogenic mixture was inoculated in the FHB. The fermentation process was carried out for 180 days, and samples were collected at different points (0, 0.5, 1, 2, 3, 5, 7, 10, 15, 30, 60, 90, 120, 150, and 180 days), and stored at 30 ± 2 °C until further analysis. The experimental batches are detailed in Table 1.

The fermented beverage’s microbial load (LAB, *E. coli, Salmonella, S. aureus*, and *B. cereus*) was assessed using the serial dilution and plate count technique [40]. MRS agar was used for culturing LAB strains. *E. coli*, *Salmonella*, *S. aureus*, and *B. cereus* were cultured in a selective medium such as Eosin Methylene Blue (EMB) agar, *Salmonella Shigella* (SS) agar, Baird–Parker agar, and Phenol Red Egg Yolk Polymyxin agar, respectively. The culture plates were incubated at 37 °C for 24 h. The colonies were counted and expressed as log CFU/mL. The changes in pH during fermentation were measured using a pH meter at room temperature.

### 2.8. Statistics Analysis

All experiments were performed in triplicate. The analytical data were expressed as mean ± standard deviations. The data were analyzed with analysis of variance (ANOVA) using SPSS (version 17, SPSS Inc., Chicago, IL, USA). Tukey tests were used to determine significant differences between the mean values at a significant level of 95% (*p* < 0.05).

## 3. Results

### 3.1. Microbial Growth of E. faecium OV3-6 Count in MRS Medium

**Figure 1:**
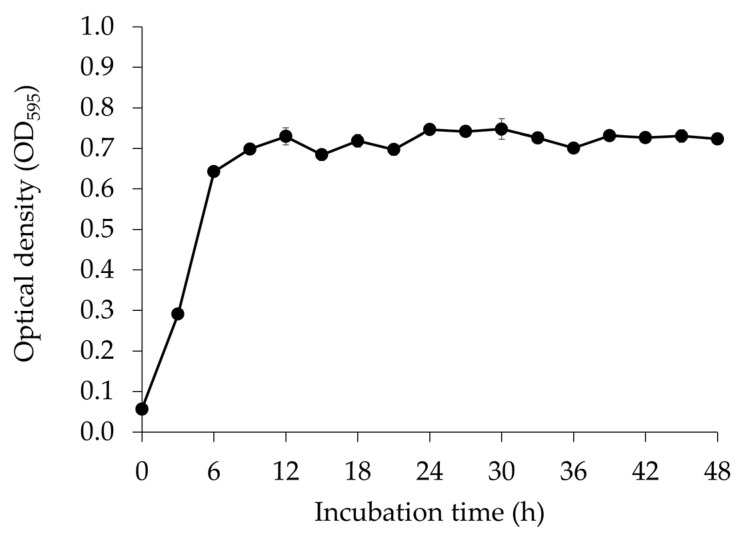
The growth (represented in OD) of *E. faecium* OV3-6 in MRS broth. Error bars represent the standard deviation of experimental triplicates.

*E. faecium* OV3-6 count in MRS medium was assessed to find out the growth curve of the strain and represented as OD. The microbial growth was rapid, and the OD reached 0.70 ± 0.005 after 9 h of culturing from 0.06 ± 0.002. The growth was relatively stable after 9 h. The maximum OD of 0.75 ± 0.003 was observed after 30 h of incubation (Figure 1).

### 3.2. pH Change during the Growth of E. faecium OV3-6 in MRS Medium

**Figure 2:**
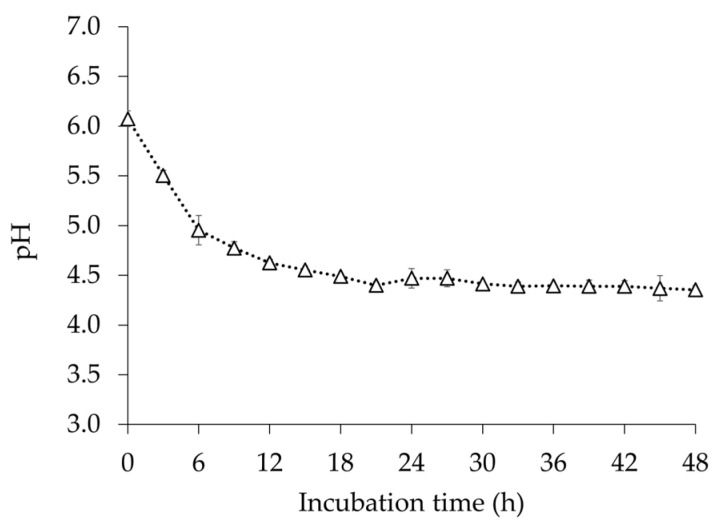
The changes in the pH of MRS broth during *E. faecium* OV3-6 growth. Error bars represent the standard deviation of experimental triplicates.

The assessment of pH change provides information about the utilization of sugars in the medium and fermentation. pH of the medium during *E. faecium* OV3-6 cultivation was exhibited in the range of 4.34 ± 0.04 to 6.08 ± 0.08. During *E. faecium* OV3-6 cultivation, the pH value of the medium gradually decreased. The lowest (4.34 ± 0.04) and highest (6.08 ± 0.08) pH values were observed at 48 and 0 h of the cultivation, respectively (Figure 2).

### 3.3. Antimicrobial Activity of E. faecium OV3-6

**Figure 3:**
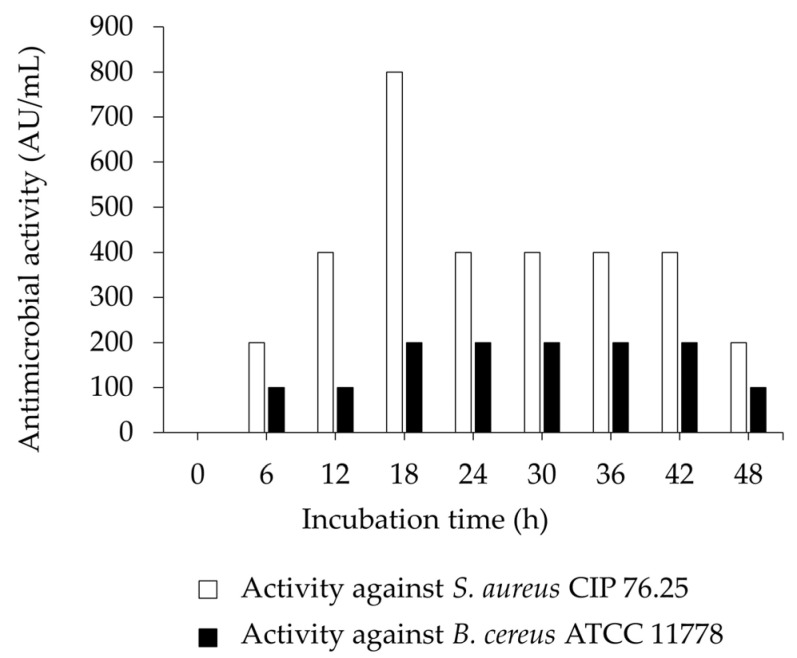
Antimicrobial activity (represented as AU/mL) of *E. faecium* OV3-6 against selected pathogens. Error bars represent the standard deviation of experimental triplicates.

The antimicrobial activity was presented as a growth inhibitory property of *E. faecium* OV3-6 against *S. aureus* CIP 76.25 and *B. cereus* ATCC 11778 (Figure 3). The highest inhibitory activity against *S. aureus* CIP 76.25 and *B. cereus* ATCC 11778 was found to be 800 AU/mL (at 18 h of incubation time) and 200 AU/mL (at 18, 24, 30, 36, and 42 h of incubation time), respectively. The lowest inhibitory activity was 200 AU/mL at 6 and 48 h for *S. aureus* CIP 76.25 and 100 AU/mL at 6, 12, and 48 h for *B. cereus* ATCC 11778. There were no changes observed in the experimental triplicates.

### 3.4. Cell Number of E. faecium OV3-6 in FB with and without H. cordata

**Figure 4:**
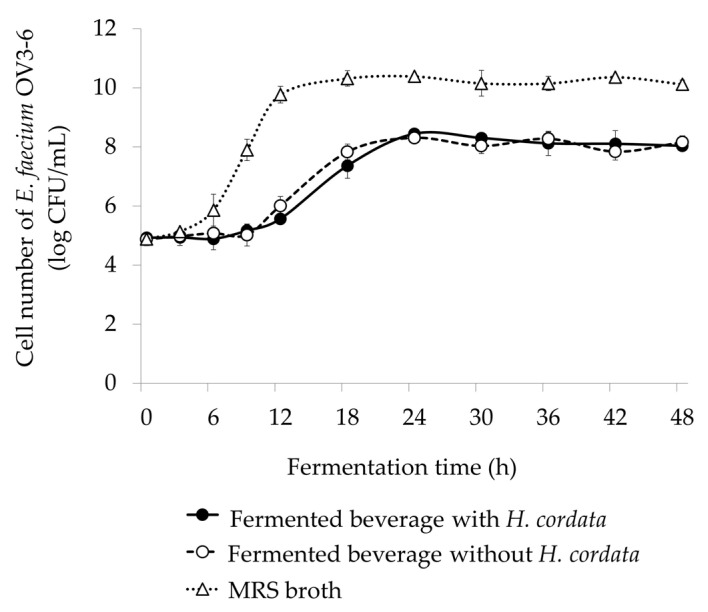
The growth of *E. faecium* OV3-6 in fermented beverage (FB) with and without *H. cordata* and MRS broth. Error bars represent the standard deviation of experimental triplicates.

The measurement of the cell number of *E. faecium* helps to know whether the strain is growing appropriately without any inhibitions from the plant material used. The alterations of bacterial cell numbers in FBs with and without *H. cordata* were exhibited during 48 h of fermentation time (Figure 4). Cell counts ranged from 4.92 ± 0.17 to 8.44 ± 0.05 log CFU/mL in FB with *H. cordata* and 4.87 ± 0.22 to 8.31 ± 0.09 log CFU/mL in FB without *H. cordata*.

The cell number of *E. faecium* OV3-6 rapidly increased during 9–24 h of fermentation in FBs with and without *H. cordata*. The maximum cell number of FBs with and without *H. cordata* was observed after 24 h of fermentation. After 24 h of fermentation, the *E. faecium* OV3-6 cell number trend was stable in both beverages (Figure 4). *E. faecium* OV3-6 was cultured in MRS broth and served as a control. The result showed that the *E. faecium* OV3-6 cell number gradually increased and reached 10.39 ± 0.08 log CFU/mL after 24 h of incubation; then, there was no significant change in the cell number (Figure 4).

### 3.5. Microbial Load in FHBs with Different Preservative Treatments

**Figure 5:**
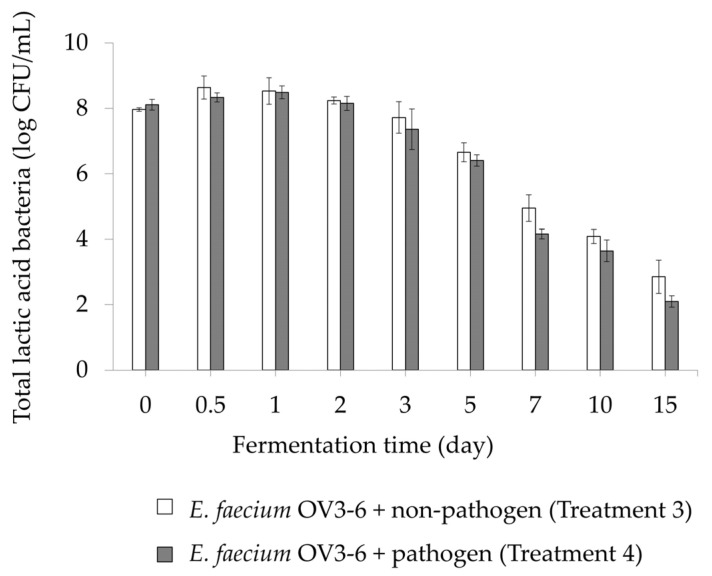
The lactic acid bacteria (LAB) profile in FHBs with different preservatives. LAB was not detected in treatments 1, 2, 5–8. Also, LAB was not detected in any treatments after 15 days of fermentation.

The analysis of microbial load provides the microbial content of the FHB. The LAB load of treatments 3 and 4 were 2.85 ± 0.51 to 8.64 ± 0.35 log CFU/mL and 2.10 ± 0.17 to 8.49 ± 0.20 log CFU/mL, respectively. The maximum number of total LAB in treatments 3 and 4 were observed at day 0.5 (8.64 ± 0.35 log CFU/mL) and day 1 (8.49 ± 0.20 log CFU/mL), respectively. Then, the growth gradually decreased until day 15 (Figure 5).

Total *E. coli* in FHBs with different preservatives was studied. On day 0, the total *E. coli* in treatments 2, 4, 6, and 8 were 5.17 ± 0.35, 5.57 ± 0.06, 5.12 ± 0.42, and 5.07 ± 0.10 log CFU/mL, respectively. After 12 h (0.5 days), no *E. coli* were detected in treatments 4 and 6. The total *E. coli* in treatments 2 and 8 ranged from 4.85 ± 0.17 to 7.69 ± 0.08 log CFU/mL and 4.31 ± 0.39 to 8.02 ± 0.14 log CFU/mL, respectively. The maximum load of *E. coli* was found after 0.5 days of the fermentation process in treatments 2 and 8. Then, a gradual reduction was observed, and after 7 days, no *E. coli* were detected (Table 2). The total *Salmonella* in treatments 2 and 8 were 8.47 ± 0.31 log CFU/mL and 3.04 ± 0.04 to 8.23 ± 0.18 log CFU/mL, respectively. The maximum load of *Salmonella* was found during 0.5 to 1st day of the fermentation process in treatments 2 and 8. Then, a gradual reduction was observed, and after 15 days, no *E. coli* was detected. Initially (0 days), in treatments 4 and 6, 5.57 ± 0.06 and 5.10 ± 0.44 log CFU/mL of *Salmonella* was detected, respectively, then it was not detected. The initial observation of *E. coli* and *Salmonella* in treatments 4 and 6 was due to the forced inoculation of the pathogen in the fermentation medium (Table 2).

Initially, the total *S. aureus* in treatments 2, 4, 6, and 8 were 5.47 ± 0.26, 5.06 ± 0.09, 5.55 ± 0.07, and 5.32 ± 0.22 log CFU/mL, respectively. While total *B. cereus* in treatments 2, 4, 6, and 8 were 5.30 ± 0.06, 5.17 ± 0.20, 5.09 ± 0.27, and 5.25 ± 0.21 log CFU/mL, respectively. After 12 h (0.5 days), no *S. aureus* and *B. cereus* were detected. The amount of *S. aureus* and *B. cereus* in the different treatments was not significantly different. Also, the total LAB, *E. coli, Salmonella, S. aureus*, and *B. cereus* were not detected in treatments 1, 3, 5, and 7 during the fermentation, indicating that the fermentation processes were performed in sterile conditions (Table 2).

### 3.6. pH Changes in FHB with Different Preservative Treatments

**Figure 6:**
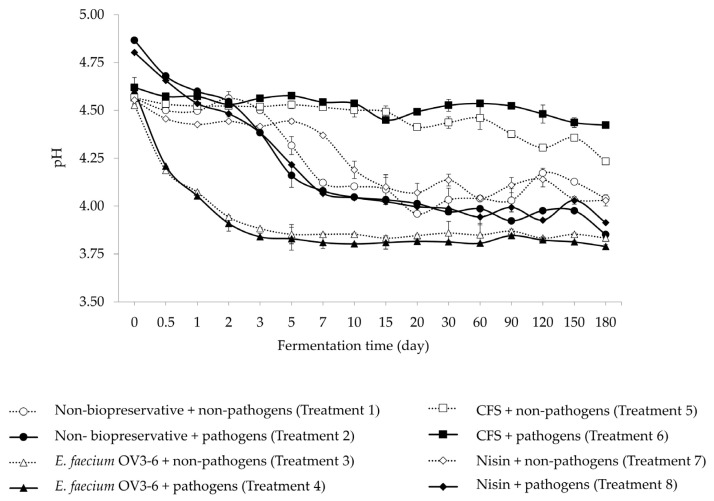
The change in pH values in FHBs with different preservatives.

The changes in pH of the different preservative treatments in FHBs are displayed in Figure 6. The treatments with pathogens showed high pH compared to non-pathogenic treatments. The pH profile of all experimental treatments guardedly decreased during 180 days of fermentation. After the fermentation process (180 days), treatments 5 (4.23 ± 0.01) and 6 (4.42 ± 0.01) showed high pH, whereas treatments 3 (3.83 ± 0.01) and 4 (3.79 ± 0.01) showed relatively low pH.

## 4. Discussion

The growth of *E. faecium* OV3-6 in MRS broth was measured (Figure 1), and the pH was reduced from 6.08 to 4.63 (Figure 2). During the growth, the strain could convert glucose to lactic acid through homofermentative pathways [41,42]. The decrease in pH and increase in acidity were more pronounced during dextrose fermentation, likely due to the higher production of lactic acid. When a probiotic strain grows in MRS broth, pH changes in the culture medium are primarily caused by the production of organic acids, especially lactic acid. Various factors, including unique characteristics of the bacterial strain and the composition of the medium, can influence the rate and extent of these pH changes. Monitoring these pH changes is essential for understanding how probiotic strains grow and metabolize nutrients, and it helps optimize the conditions for their cultivation [43,44].

The lower pH may provide suitable ionic strength; after a point, the pH reduction could inhibit the bacteria’s growth [45,46]. Many studies have reported the relationship between bacterial growth and bacteriocin production [47,48]. Bacteriocin-like inhibitory substances from Gram-positive bacteria (BGPBs) exhibit antimicrobial properties primarily by disrupting the structural integrity of bacterial membranes. These antimicrobial peptides employ diverse mechanisms to achieve this goal. For instance, certain BGPBs, like nisins, interact directly with lipid II, a specific component of the bacterial membrane, inducing the formation of pores. These pores compromise membrane integrity, leading to increased permeability and cell death. In contrast, microbisporicin disrupts the bacterial cell wall biosynthesis process, accumulating cell wall precursors within the bacterial cell. This accumulation perturbs the membrane and contributes to cell demise. Class II bacteriocins, including pediocin PA-1, target the Man-PTS system in bacteria, causing constant receptor opening and uncontrolled efflux of intracellular molecules, leading to cell damage. Subclass IIb bacteriocins such as lactococcin Q and lactococcin G are thought to create membrane pores, potentially through interactions with specific membrane proteins. In summary, BGPBs employ a range of mechanisms to destabilize bacterial membranes or disrupt essential bacterial processes, culminating in the death of the targeted bacteria [49,50,51,52,53,54]. Regarding BGNB (Bacteriocin-Producing Gram-Negative Bacteria) bacteriocins, microcins demonstrate their antibacterial effects through one of two distinct mechanisms: (i) the creation of pores within the inner bacterial membrane, as seen with microcins E492, M, and H47; (ii) the targeting of intracellular enzymes, exemplified by microcin J25, B17, and C. Unlike BGPBs, microcins necessitate entry into the targeted bacterial cell to exert their antimicrobial activity. Once inside, they utilize specific receptors located on the outer membrane of susceptible strains. These receptors are associated with iron uptake and outer membrane porin functions [54,55,56].

Valledor et al. [57] and Barman et al. [58] observed that a high level of bacteriocin production occurred at the late log phase and early stationary phase. The bacteriocins are microbial secondary metabolites, supplementary compounds produced by the microbes for interaction or competition with other organisms or the environment [59]. The early stationary phase of microbial growth is optimal for producing bacteriocin by probiotic strains [60]. 

Favaro et al. [61] studied the production of bacteriocins by *E. faecium* ST209GB, ST278GB, ST315GB, and ST711GB isolated from Bulgarian homemade white brine cheese. The study suggested that bacteriocin production was observed in logarithmic growth, and a high level of bacteriocin was displayed in stationary growth. Similarly, Abdel–Hamidet et al. [33] reported the relationship between the growth curve and bacteriocin production in *E. faecium* ER-3M (isolated from cattle raw milk). The peak of bacteriocins activity was observed in the early stationary phase of *E. faecium* ER-3M growth.

In the present study, the highest microbial inhibition was observed against *B. cereus* ATCC 11778 and *S. aureus* CIP 76.25 after 18 h of the incubation period, which was in the range of the early stationary phase of *E. faecium* OV3-6 (Figure 3). The study found that 18 h of culturing of *E. faecium* OV3-6 in an MRS medium could provide peak bacteriocin activity. The bacteriocin of *E. faecium* OV3-6 was also noticeable at the end of the incubation time. The activity against *B. cereus* ATCC 11778 and *S. aureus* CIP 76.25 slightly decreased after 48 h incubation (Figure 3). The proteolytic degradation of bacteriocin may be related to the reduction in its bioactivity after 48 h [62]. The enzymes could destroy the bacteriocin that *E. faecium* OV3-6 produces during incubation. In addition, some literature reported that the loss of bacteriocin is related to the reabsorption to the surface of the producer cell at a low pH [63].

It is necessary to confirm that the bio-active properties of raw materials should not affect the starter cultures used for the fermentation process. An essential oil isolated from the aerial parts and underground stem of *H. cordata* showed antimicrobial activities against *S. aureus*, *Streptococcus mutans*, *Mycobacterium smegmatis*, *E. faecalis*, *Candida albicans*, and *Candida kefyr* [11]. Sekita et al. [10] demonstrated that the ethanolic extract of *H. cordata* poultice showed antimicrobial activity against Gram-positive bacteria, such as *Streptococcus epidermidis*, *Streptococcus pyogenes*, *Streptococcus mitis*, and *E. faecalis*. 

In the present study (Figure 4), the cell number of *E. faecium* OV3-6 was higher in MRS broth, which acted as a control since the MRS broth has all the essential nutrients for bacteria growth [64,65]. The trend in changes in cell number of *E. faecium* OV3-6 cultured in FBs with and without *H. cordata* was the same until 48 h of fermentation time, which indicated that *H. cordata* could be used as a raw material to produce fermented beverages using *E. faecium* OV3-6 as a starter culture. 

Many bio-preservative methods have been used to enhance safety and extend the shelf life of food products using natural agents [66]. LAB is used as a natural preservative in various food products due to its antimicrobial (via bacteriocins) and metabolic activity (hydrogen peroxide and lactic acid) [67]. LAB bacteriocins are considered acceptable bio-preservative agents because they are non-toxic, non-immunogenic, thermo-resistant, and have extensive bactericidal activity [68,69].

The most commercial LAB bacteriocin is nisin [28,70], which is widely used in the food industry. Nisin is safe, and its use has been approved by the Food and Drug Administration [24,27,30]. Thus, we used nisin as one of the bio-preservative agents in FHBs.

Treatments 3 and 4 showed their LAB content until 15 days of fermentation. In the presence of pathogens, the growth of *E. faecium* OV3-6 was not affected in FHBs (Figure 5). The study reported that bacteriocin-producing *L. plantarum* LPL-1 could change the microbial community in low-salt fermented sausages [21]. However, LAB’s efficiency in fermentation depends on various factors such as nutrient composition, time, pH, temperature, material ingredients, and antagonistic microorganisms [71,72]. Additionally, most LAB strains are probiotics with health-promoting properties [73]. We previously reported that *E. faecium* OV3-6 has in vitro probiotic activity [32].

The pathogenic strains (*E. coli*, *Salmonella*, *S. aureus*, and *B. cereus*) were observed in FHBs on day 0. Total *E. coli* was observed during 0–7 days of the fermentation period. Total *Salmonella* was observed during 0–15 days of the fermentation period. Gram-positive pathogens (*S. aureus* and *B. cereus*) were not detected after 0.5 days of fermentation (Table 2). Treatments with *E. faecium* OV3-6 (treatment 4) and CFS of *E. faecium* OV3-6 (treatment 6) could have bacteriocin, which inhibits both Gram-positive and Gram-negative pathogenic bacteria. 

Waheed et al. [74] reported that the growth of Gram-negative pathogens, *E. coli* SABA3 and *S. typhi* SABA10, was reduced by 85% and 50%, respectively, by *E. faecium* SANA1. Anyogu et al. [75] also suggested that CFS of *E. faecium* CL02 was able to inhibit both Gram-positive pathogenic bacteria (*B. cereus* and *S. aureus*) and Gram-negative pathogenic bacteria (*E. coli* and *S. typhimurium*) in vitro. Mostly, bacteriocins destroy Gram-positive bacteria by inhibiting peptidoglycan synthesis and pore formation in the cell membrane, resulting in a loss of potential membrane and cell death [76]. Some bacteriocins control the Gram-negative bacterial growth by pore formation in the cell membrane and enzyme interference, such as DNA gyrase, RNA polymerase, and aspartyl-tRNA synthetase [77].

Treatment 2 (non-preservative) and treatment 8 (with nisin) inhibited the Gram-positive pathogens throughout fermentation. Nisin inhibits Gram-positive pathogens via its action on the outer peptidoglycan layer of Gram-positive bacteria [78,79]. It has been proven that medicinal plants have antimicrobial properties against Gram-positive pathogens [10,80,81]. Accordingly, the antimicrobial effects against Gram-positive pathogens (*B. cereus* and *S. aureus*) in treatment 2 (non-preservative) are possibly due to the antimicrobial activity of the raw material (*H. cordata*) used in the study (Table 2). Treatment 8 (with nisin) showed antimicrobial activity against Gram-positive pathogens [30,82] but not against Gram-negative bacteria due to the protective outer membrane of the bacterium [83]. Therefore, nisin has some limitations; *E. faecium* OV3-6 and its metabolites are effective alternative bio-preservatives to produce fermented FHBs.

The pH level could indicate the safety and quality of the fermented product. According to Thai Community Product Standard (TCPS)—No. 481/2004, the acceptable pH level in FPBs is less than 4.3 [84]. In this study, treatments 3 and 4 (with *E. faecium* OV3-6) showed a pH value of less than 4.3 after 0.5 days (Figure 6). Similarly, Liu et al. [37] reported that the pH of Paocai (lactic acid-fermented vegetable), which uses *E. faecium* Y31 as an adjunct culture, was less than 4.3 after 2.5 days of fermentation. The FHBs with non-preservative *E. faecium* OV3-6 and nisin (treatments 1, 2, 3, 4, 7, and 8) were in the pH range of TCPS acceptance level at the end of fermentation. A few studies reported that the production of organic acids (lactic and acetic acids) could affect the pH of the fermented product [85,86]. However, treatments 5 and 6 (containing CFS of *E. faecium* OV3-6) showed a pH higher than 4.3 throughout fermentation (Figure 6).

Many reports have indicated that the metabolites in CFS of LAB have efficacy in inhibiting microbial pathogens [87,88,89]. The secreted metabolites in CFS of *E. faecium* OV3-6 might inhibit the growth of the other epiphytic microflora [90,91,92], which might affect organic acid production. Thus, the pH value of treatments 5 and 6 was substantially higher than the other treatments. The above statements need to be confirmed through detailed studies.

This study has some limitations, as detailed below. Even though we reported the presence of the *entP* gene in *E. faecium* OV3-6 that involves the production of bacteriocin called enterocin P, further detailed characterization of the bacteriocins-like substances was not studied. The metabolomic profile may provide detailed information on nutritional changes of the FHB, but the present study lacks that. Also, studies are required to determine the effect of *H. cordata* on the bacteriocin production or microbial activity of *E. faecium* OV3-6. The in vitro and in vivo evaluation of bioactivities and health effects of FHBs is necessary to claim that FHBs are functional food. Finally, the consumer acceptance survey also required us to market FHBs.

## 5. Conclusions

The study showed that *E. faecium* OV3-6 could produce bacteriocin-like substances during its late log phase and the early stationary phase in MRS broth. Maximum bacteriocin-like substances were produced after 18 h of incubation in MRS broth, which was confirmed through the antimicrobial activity against the representative pathogens. The survival of *E. faecium* OV3-6 in FBs with and without *H. cordata* indicated that *E. faecium* OV3-6 could be potent starter culture to produce FHBs. The pH value of FHBs with *E. faecium* OV3-6 was in the acceptable range (less than 4.3). The LAB load in FHBs was higher during fermentation with *E. faecium* OV3-6; it could be considered that the strain *E. faecium* OV3-6 improved the LAB and probiotics in FHBs. The preliminary study results indicated that *E. faecium* OV3-6 and CFS of *E. faecium* OV3-6 could be bio-preservatives to control undesirable pathogens in FPBs. Biobased preservatives could be the better option to protect customers from the adverse side effects of chemicals.

The study has some limitations, including the safety of the FHB not being studied, changes in the metabolomics were not assessed in the FHB, and the health effects of the FHB were not studied using laboratory models. Also, the FHB needs to be evaluated for consumer acceptance. These limitations block the strong claim that *E. faecium* OV3-6 is a potent bio-preservative to produce FHBs. Further extensive studies on the abovementioned aspects of FHBs are needed to overcome the restrictions in *E. faecium* OV3-6-based bio-preservatives.

## Figures and Tables

**Table 1:** Experimental setup with or without preservatives in FHBs.

Treatment Numbers	Factors	
	Bio-Preservatives	Pathogens *
1	No preservative	-
2	No preservative	+
3	*E. faecium* OV3-6	-
4	*E. faecium* OV3-6	+
5	CFS of *E. faecium* OV3-6	-
6	CFS of *E. faecium* OV3-6	+
7	Nisin	-
8	Nisin	+

* A mixture of *E. coli* ATCC 5922, *S. typhi* DMST 22842, *S. aureus* CIP 76.25, and *B. cereus* ATCC 11778; at the concentration of 10^7^ CFU/mL each strain; +: Presence of pathogens; -: Absence of pathogens.

**Table 2:** The representative pathogens load during the fermentation of FHB with different preservatives.

Treatments	*E. coli* (Log CFU/mL)								
	0 Day	0.5 Day	1 Day	2 Days	3 Days	5 Days	7 Days	10 Days	15 Days
2	5.17 ± 0.35 ^a^	7.69 ± 0.08 ^a^	7.43 ± 0.01 ^a^	7.30 ± 0.15 ^b^	6.91 ± 0.13 ^b^	6.03 ± 0.40 ^a^	4.85 ± 0.17 ^b^	ND	ND
4	5.57 ± 0.06 ^a^	ND	ND	ND	ND	ND	ND	ND	ND
6	5.12 ± 0.42 ^a^	ND	ND	ND	ND	ND	ND	ND	ND
8	5.07 ± 0.10 ^a^	8.02 ± 0.14 ^b^	7.81 ± 0.18 ^b^	6.37 ± 0.39 ^a^	6.03 ± 0.02 ^a^	5.72 ± 0.01 ^a^	4.31 ± 0.39 ^a^	ND	ND
	Total *Salmonella* (Log CFU/mL)								
2	5.18 ± 0.15 ^a^	8.47 ± 0.31 ^b^	8.22 ± 0.01 ^a^	6.95 ± 0.10 ^a^	6.59 ± 0.15 ^a^	6.38 ± 0.29 ^a^	6.30 ± 0.15 ^b^	5.95 ± 0.05 ^a^	2.97 ± 0.07 ^a^
4	5.57 ± 0.06 ^a, b^	ND	ND	ND	ND	ND	ND	ND	ND
6	5.10 ± 0.44 ^a^	ND	ND	ND	ND	ND	ND	ND	ND
8	5.85 ± 0.10 ^b^	8.12 ± 0.14 ^a^	8.23 ± 0.18 ^a^	6.96 ± 0.04 ^a^	6.72 ± 0.24 ^a^	6.93 ± 0.01 ^b^	5.55 ± 0.10 ^a^	6.00 ± 0.03 ^a^	3.04 ± 0.04 ^a^
	*S. aureus* (Log CFU/mL)								
2	5.47 ± 0.26 ^b^	ND	ND	ND	ND	ND	ND	ND	ND
4	5.06 ± 0.09 ^a^	ND	ND	ND	ND	ND	ND	ND	ND
6	5.55 ± 0.07 ^b^	ND	ND	ND	ND	ND	ND	ND	ND
8	5.32 ± 0.22 ^a, b^	ND	ND	ND	ND	ND	ND	ND	ND
	*B. cereus* (Log CFU/mL)								
2	5.30 ± 0.06 ^a^	ND	ND	ND	ND	ND	ND	ND	ND
4	5.17 ± 0.20 ^a^	ND	ND	ND	ND	ND	ND	ND	ND
6	5.09 ± 0.27 ^a^	ND	ND	ND	ND	ND	ND	ND	ND
8	5.25 ± 0.21 ^a^	ND	ND	ND	ND	ND	ND	ND	ND

Data are shown as means ± SD of triplicate determinations. The letters indicate that the values are significantly different (*p* ˂ 0.05) with an individual column. No microbial load was found in treatments 1, 3, 5, and 7. ND: Not detected.

## Data Availability

All the data are available within the manuscript.
